# Validation of the Arabic Language Version of the Shortest Form of the Community Assessment of Psychic Experiences (CAPE‐9) in a Young Adult Population‐Based Sample

**DOI:** 10.1111/eip.70158

**Published:** 2026-03-08

**Authors:** Feten Fekih‐Romdhane, Diana Malaeb, Fouad Sakr, Mariam Dabbous, Sahar Obeid, Souheil Hallit

**Affiliations:** ^1^ The Tunisian Center of Early Intervention in Psychosis, Department of Psychiatry “Ibn Omrane” Razi Hospital Manouba Tunisia; ^2^ Faculty of Medicine of Tunis Tunis El Manar University Tunis Tunisia; ^3^ College of Pharmacy Gulf Medical University Ajman UAE; ^4^ School of Pharmacy Lebanese International University Beirut Lebanon; ^5^ Social and Education Sciences Department, School of Arts and Sciences Lebanese American University Byblos Lebanon; ^6^ School of Medicine and Medical Sciences Holy Spirit University of Kaslik Jounieh Lebanon; ^7^ Applied Science Research Center Applied Science Private University Amman Jordan

**Keywords:** Arabic, CAPE‐9, psychometrics, psychosis risk, psychotic experiences, short form

## Abstract

**Background:**

Whilst self‐report questionnaires measuring psychotic experiences (PEs) in community individuals have recently been made available in the Arabic language, their length may be a barrier to PEs assessment in settings in lower‐to‐middle income Arab countries, that often operate under severe resources constraints. The purpose of the present study was to investigate the psychometric properties of the Arabic‐language version of the shortest version of the Community Assessment of Psychic Experiences‐Positive (CAPE‐P), i.e., the CAPE‐9, in a sample of community adults.

**Methods:**

A cross‐sectional study was carried out using a web‐based questionnaire. A total of 685 Arabic‐speaking adults from the general population of Lebanon (mean age of 23.54 ± 4.58 years, 66.0% females) participated.

**Results:**

The internal structure of the Arabic CAPE‐9 demonstrated that the overall model fit of the tridimensional factor structure (consisting of ‘Persecutory Ideation’, ‘Bizarre Experiences’, and ‘Perceptual abnormalities’ sub‐dimensions) was acceptable. A unidimensional model was tested and exhibited borderline fit indices, with all nine items loading onto one factor. Internal consistency coefficient estimates were of alpha = 0.84 and omega = 0.84 for the total score, and ranged from 0.64 to 0.70 for omega and alpha values for the three sub‐scores. Measurement invariance between males and females was established for both models, with no significant difference noticed between genders. Finally, significant positive associations were found between PEs and anxiety, depression, insomnia severity, and aggression, indicating the validity of the scale.

**Conclusion:**

As a short, simple, economic, and convenient‐to‐administer measure of PEs, the CAPE‐9 is amenable to widespread use. It has, therefore, the potential to foster research and clinical practise by easing data collection, lessening burden, and enhancing engagement of respondents. It may substantially improve recognition of a substantial proportion of undetected patients with psychotic disorders and contribute to reducing the very long duration of untreated psychosis still observed in Arab countries.

## 1 Introduction

Psychotic experiences (PEs) can be defined as endorsing unusual beliefs, thoughts, or perceptions, such as persecutory ideation, thought broadcasting, or auditory/visual hallucinations, without meeting the clinical threshold for a psychotic disorder (Linscott and Van Os 2013; McGrath et al. 2015). PEs are experienced by a significant part of the general population, particularly adolescent and young adult people (Fekih‐Romdhane, Pandi, et al. 2022; Linscott and Van Os 2013). In spite of being insufficient in frequency, severity or impairment to characterise the full‐blown psychosis, PEs are not necessarily innocuous (Murray and Jones 2012). Indeed, the psychosis continuum framework suggests that psychotic symptoms are expressed along an extended psychosis spectrum phenotype, where PEs share etiological risk factors with, and predict later psychotic disorders (Kaymaz et al. 2012; Linscott and Van Os 2013). In addition, PEs are increasingly recognised as risk markers for broader psychopathological vulnerability, poor mental health outcomes, addictive and suicidal problems, functional disability, and an elevated need for care (Fekih‐Romdhane 2024; Fekih‐Romdhane, Lamloum, Loch, et al. 2023; Fekih‐Romdhane, Malaeb, Loch, et al. 2023; Linscott and Van Os 2013; Rössler et al. 2007; Rubio et al. 2012).

As a consequence of this evidence, more attention has been devoted to the predictive value and detection of PEs as key to prevention and early intervention of both psychotic and non‐psychotic psychopathology in the general population (Bhavsar et al. 2021; Veijola et al. 2013). Subsequently, the need was raised for sound measures to timely identify and monitor the presence, impact and evolution of PEs within non‐clinical samples and offer an opportunity for preventive strategies (Kelleher, Lynch, et al. 2012). Although clinician‐administered semi‐structured interviews (such as the Comprehensive Assessment of At‐Risk Mental States [CAARMS], (Yung et al. 2005)) are gold standards, such tools are lengthy, time‐, cost‐, and resource‐consuming. In contrast, self‐report measures require limited time, costs and resources to administer, and are therefore more practical for primary screening of large populations in diverse settings, including where trained interviewers are unavailable and time constraints are present (Addington et al. 2015). One of the most well‐known and commonly used self‐report assessments for screening for PEs in the general population is the Community Assessment of Psychic Experiences positive scale (CAPE‐P; (Stefanis et al. 2002)).

### 1.1 The CAPE‐P

The CAPE‐P originally encompasses 20 items designed to assess lifetime positive PEs in terms of frequency and distress in the general population (Stefanis et al. 2002). The CAPE‐P showed good construct and discriminant validity, as well as satisfactory test–retest reliability (Addington et al. 2015; Mark and Toulopoulou 2016; Stefanis et al. 2002). Since its original development, the CAPE has been translated and validated in multiple large samples, in various languages and countries, including German (Schlier et al. 2015), Italian (Armando et al. 2012), Swedish (Ziermans 2013), French (in Canada (Brenner et al. 2007) and France (Verdoux et al. 2003)), Spanish (Fonseca‐Pedrero et al. 2012), Portuguese (Ragazzi et al. 2020), Korean (Sim et al. 2023), Indonesian (Jaya 2017), Chinese (Mark and Toulopoulou 2017), Persian (Mirzaei Poueenak et al. 2022), and Arabic (Fekih‐Romdhane, Farah, Malaeb, et al. 2023). A review and meta‐analysis by Mark and Toulopoulou (Mark and Toulopoulou 2016) comprehensively explored psychometric characteristics of the CAPE across 15 different countries, and found that its factorial validity was satisfactory, and that high reliability coefficients were obtained for general population samples in the majority of studies included. Authors concluded to the robust psychometric properties of the CAPE, and recommend it for use to measure the psychosis proneness phenotype in young populations in both clinical practise and research (Mark and Toulopoulou 2016). In addition, evidence for identical factorial structure, factor loadings and thresholds of the CAPE across six countries (i.e., UK, Italy, Spain, France, the Netherlands, and Brazil) was provided, thus allowing for cross‐country comparisons to be made (Pignon et al. 2019).

The CAPE has also demonstrated high predictive power for the onset of psychotic disorders (Welham et al. 2009) and other detrimental mental health outcomes (Brenner et al. 2007; Yung et al. 2009), thus resulting in its widespread use globally for clinical and research purposes, in both clinical and non‐clinical settings and populations (Mark and Toulopoulou 2016). Indeed, although the CAPE was mostly adopted amongst general population samples, its usefulness in clinical populations has also been demonstrated (e.g., in patients with psychosis (Fonseca‐Pedrero et al. 2012), in nonpsychotic help‐seekers (Yung et al. 2006)). The CAPE showed high sensitivity and specificity for the detection of misdiagnosed patients with first‐episode psychosis at their first contact with mental health services (Boonstra et al. 2009), as well as ability to discriminate between individuals with a diagnosis of a psychotic disorder and healthy controls (Jaya et al. 2021). It has also proven validity and effectiveness for specific assessment and detection of individuals at ultra‐high risk (UHR) for psychosis (Mossaheb et al. 2012; Williams et al. 2022).

### 1.2 Shortened Versions of the CAPE‐P

Based on the original CAPE‐P, a 15‐question version (CAPE‐P15) was firstly validated in a large sample of university students aged 18–25 years, where it revealed a three‐factor structure (i.e., persecutory ideation, bizarre experiences, and perceptual abnormalities), good validity and high levels of reliability (Capra et al. 2013). Later psychometric studies have confirmed the robustness of the scale in different populations (e.g., Chinese (Therman and Ziermans 2016), Korean (Kim et al. 2020), and Australian (Capra et al. 2017) university students, Chilean adolescents (Núñez et al. 2015)) in terms of concurrent and construct validity, internal consistency, test–retest reliability, and measurement invariance across sex. Evidence was also provided for its utility in assessing PEs both over a lifetime and in the past month (Capra et al. 2017; Therman and Ziermans 2016), its accuracy in classifying and differentiating adolescents from the general population according to their PEs severity levels (Núñez et al. 2021), and its effectiveness in identifying UHR individuals in clinical settings (Bukenaite et al. 2017). More recently, a newer short version of the CAPE‐P15 has been introduced and validated in a Population‐Based Sample of 29 021 Norwegian Adult Men (Birkenæs et al. 2023). Authors indicated that the CAPE‐9 is an effective tool, with a supported 3‐factor structure comparable to longer versions and an acceptable to good reliability (Birkenæs et al. 2023). In addition, CAPE‐9 scores were significantly associated with schizophrenia and trauma‐related disorders, thus suggesting its usefulness as a cost‐effective tool for measuring lifetime PEs in the adult general population (Birkenæs et al. 2023). However, due to its recent development and validation, a comprehensive investigation of psychometric characteristics of this briefest form of the CAPE‐P in both male and female people, and other‐than‐Western cultural contexts, has yet to be performed.

### 1.3 The Present Study

There is some agreement amongst researchers towards developing and using shorter scales that reduce the burden on respondents and save time, whilst maintaining the desirable qualities of the corresponding full‐length versions [e.g., (de Lins Holanda Coelho et al. 2020; Monteiro et al. 2022)]. Whilst self‐report questionnaires measuring PEs in community individuals have recently been made available in the Arabic language (Fekih‐Romdhane, El Hadathy, González‐Nuevo, et al. 2023; Fekih‐Romdhane, Farah, Malaeb, et al. 2023; Fekih‐Romdhane, Jahrami, Alhuwailah, et al. 2023), their length may be a barrier to PEs assessment in settings in lower‐to‐middle income Arab countries, that often operate under severe resources constraints. As such, using short self‐report tools could encourage the introduction of wide routine screening for PEs in primary care, educational, and community settings in Arab countries, where the duration of untreated illness remains incredibly high (e.g., up to around 6 years in patients with first episode psychosis in Tunisia (Fekih‐Romdhane, Abassi, Ghrissi, et al. 2023)), and where early intervention in psychosis paradigm is still in its infancy or not yet developed (Fekih‐Romdhane et al. 2023k). Therefore, the purpose of the present study was to investigate the psychometric properties of the Arabic‐language version of the shortest version of the CAPE‐P to date, i.e., the CAPE‐9, in a sample of community adults. It is hypothesised that (1) analyses will support the three‐factor structure proposed by Birkenæs et al. (Birkenæs et al. 2023), (2) the Arabic CAPE‐9 will show good internal consistency and measurement invariance across sex, (3) the scale will reveal correlations in the expected direction with measures of psychological distress, aggressiveness and insomnia, indicating concurrent validity.

## 2 Materials and Methods

### 2.1 Sample and Procedures

A cross‐sectional study was carried out during the period from July to August 2022. Eligible participants were young adults from the general population of Lebanon, aged 18–35 years, with no personal history of psychosis or antipsychotic medication intake. A web‐based survey advertised on social media was used to collect data following the snowball sampling technique. The survey was anonymous and participants completed the survey voluntarily and without remuneration. Ethics approval for this study was obtained from the School of Pharmacy at the Lebanese International University ethics committee.

### 2.2 Measures

#### 2.2.1 Socio‐Demographic Data

Age, sex, marital status, and educational level were collected for all participants.

#### 2.2.2 The Community Assessment of Psychic Experiences (CAPE‐9)

The CAPE‐9 (Birkenæs et al. 2023) is a shortened version of the CAPE‐P15 (Capra et al. 2013) and the CAPE‐42 (Stefanis et al. 2002). It assesses both the frequency and distress levels of PEs. PE frequency is indicated through a four‐point Likert scale ranging from 1 (‘never’) to 4 (‘nearly always’). For each PE, a distress score is also rated on a four‐point Likert scale from 1 (‘not distressed’) to 4 (‘very distressed’). The Arabic translated items were extracted from the version of the CAPE‐42 that has been recently validated into Arabic (the translation procedure can be found in (Fekih‐Romdhane, Farah, Malaeb, et al. 2023)).

#### 2.2.3 The Patient Health Questionnaire 9 Items (PHQ‐9)

This scale is composed of nine items, rated on a 4‐point Likert scale (0 = never to 3 = every day), with higher scores reflecting higher depression. This scale is validated in Arabic (Sawaya et al. 2016) (*ω* = 0.91/*α* = 0.91).

#### 2.2.4 The Generalised Anxiety Disorder 7 Items (GAD‐7)

This scale is composed of seven items, rated on a 4‐point Likert scale (0 = never to 3 = every day), with higher scores reflecting higher depression. This scale is validated in Arabic (Dagher et al. 2023; Sawaya et al. 2016) (*ω* = 0.92/*α* = 0.92).

#### 2.2.5 The Insomnia Severity Index (ISI)

This scale is self‐administered, and evaluates the nature, intensity, and effects of insomnia through the following seven items: sleep maintenance, sleep dissatisfaction, severity of sleep onset, distress caused by the sleep difficulties, interference of sleep difficulties with daytime functioning, early morning awakening problems, and noticeability of sleep problems by others (Bastien et al. 2001). Greater scores indicate more severe insomnia. The Arabic validated version of the ISI was adopted (Hallit et al. 2019) (*ω* = 0.87/*α* = 0.86).

#### 2.2.6 The Buss‐Perry Aggression Questionnaire‐Short Form (BPAQ‐SF)

This scale contains 12 items and four subscales measuring levels of aggression: Physical Aggression, Verbal Aggression, Anger, and Hostility (Bryant and Smith 2001). Higher scores reflect higher aggression. The Arabic validated version was used (Fekih‐Romdhane et al. 2023b). Cronbach alpha values of the BPAQ‐SF scores in our samples were the following: physical aggression (*ω* = 0.78/*α* = 0.76), verbal aggression (*ω* = 0.75/*α* = 0.72), anger (*ω* = 0.81/*α* = 0.81), and hostility (*ω* = 0.84/*α* = 0.83).

### 2.3 Analytic Strategy

#### 2.3.1 Confirmatory factor analysis (CFA)

There were no missing responses in the dataset. We used data from the total sample to conduct a CFA using the SPSS AMOS v.29 software. The minimum sample size to conduct a confirmatory factor analysis ranges from 3 to 20 times the number of the scale's variables (Mundfrom et al. 2005). Therefore, we assumed a minimum sample of 180 participants needed to have enough statistical power based on a ratio of 20 participants per one item of the scale, which was exceeded in our sample. Our intention was to test the original model of the CAPE‐9 scores. Parameter estimates were obtained using the maximum likelihood method and fit indices. To check if the model was adequate, several fit indices were calculated: the Steiger‐Lind root mean square error of approximation (RMSEA), the Tucker‐Lewis Index (TLI), and the comparative fit index (CFI). Values ≤ 0.08 for RMSEA, ≤ 0.05 for SRMR, and 0.90 for CFI and TLI indicate good fit of the model to the data (Hu and Bentler 1999). Multivariate normality was not verified at first; therefore, we performed a non‐parametric bootstrapping procedure. Specifically, bootstrapping was performed using 2000 resamples with bias‐corrected 95% confidence interval estimated for all standardised loading factors. The bootstrapped CI did not substantially differ from the original maximum likelihood estimates, and all factor loadings remained statistically significant indicating the stability of the parameter estimates. In addition, model fit indices derived from the bootstrapped samples were comparable to those obtained from the primary analysis, supporting the robustness of the CFA results despite deviations from multivariate normality and the demographic skew of the sample, particularly the overrepresentation of females.

To examine sex invariance of the CAPE‐9 scores, we conducted multi‐group CFA (Chen 2007) using the total sample. Measurement invariance was assessed at the configural, metric, and scalar levels (Vadenberg and Lance 2000). We accepted ΔCFI ≤ 0.010 and ΔRMSEA ≤ 0.015 or ΔSRMR ≤ 0.010 as evidence of invariance (Swami et al. 2022).

Internal reliability was assessed using McDonald's *ω* and Cronbach's alpha, with values greater than 0.70 reflecting adequate internal reliability (Dunn et al. 2014). The CAPE‐9 scores were not normally distributed according to their skewness and kurtosis values varying outside the ±1 range (Hair Jr et al. 2017); therefore, the log transformation was applied, which yielded a normally distributed score. Consequently, the Pearson test was used to correlate scores between each other and the Student *t*‐test was used to compare two means.

## 3 Results

The total sample was composed of 685 Lebanese young adults, with a mean age of 23.54 ± 4.58 years, 66.0% females, 81.9% single, and 81.3% with a university level of education. Other description of the sample can be found in Table 1.

**TABLE 1:** Sociodemographic and other characteristics of the participants (*n* = 685).

Sex	
Male	233 (34.0%)
Female	452 (66.0%)
Marital status	
Single	561 (81.9%)
Married	124 (18.1%)
Education	
Secondary or less	128 (18.7%)
University	557 (81.3%)

	Mean ± SD
Age (in years)	23.54 ± 4.58
CAPE‐9	4.13 ± 4.09
Physical aggression	6.47 ± 3.05
Verbal aggression	6.59 ± 2.99
Anger	6.89 ± 3.36
Hostility	6.60 ± 3.26
Insomnia severity	8.34 ± 5.85
Anxiety	6.06 ± 5.15
Depression	7.08 ± 6.16

### 3.1 Confirmatory Factor Analysis of the CAPE‐9

CFA indicated that fit of the three‐factor model of the CAPE‐9 scale was acceptable: RMSEA = 0.074 (90% CI 0.060, 0.088), SRMR = 0.041, CFI = 0.950, TLI = 0.925. The internal reliability of the total and subscales scores was borderline as follows: persecutory ideation (*ω* = 0.70/*α* = 0.69), bizarre experiences (*ω* = 0.64/*α* = 0.64) and perceptual abnormalities (*ω* = 0.70/*α* = 0.70).

Fit of the one‐factor model of the CAPE‐9 scale was borderline: RMSEA = 0.082 (90% CI 0.070, 0.095), SRMR = 0.046, CFI = 0.930, TLI = 0.906 (*ω* = 0.84/*α* = 0.84). The standardised loading factors for both models are summarised in Table 2.

**TABLE 2:** English items of the CAPE‐9, their frequency, and standardised estimates of factor loadings from the confirmatory factor analysis for the one‐ and three‐factor models.

	Frequency of each item				Associated distress				3 factors	1 factor
	Never	Sometimes	Often	Nearly always	Not distressed	A bit distressed	Quite distressed	Very distressed		
Delusions of reference	333 (48.6%)	300 (43.8%)	44 (6.4%)	8 (1.2%)	485 (70.8%)	171 (25.0%)	24 (3.5%)	5 (0.7%)	0.57	0.56
*‘Have you ever felt that what is printed in magazines and newspapers or said on TV specifically applies to you?’*										
2 Beliefs about stalking	431 (62.9%)	214 (31.2%)	27 (3.9%)	13 (1.9%)	452 (66.0%)	156 (22.8%)	56 (8.2%)	21 (3.1%)	0.74	0.68
*‘Have you ever felt that someone is stalking you in some way?*’										
3 Beliefs about conspiracy	458 (66.9%)	176 (25.7%)	41 (6.0%)	10 (1.5%)	481 (70.2%)	137 (20.0%)	51 (7.4%)	16 (2.3%)	0.67	0.62
‘*Have you ever felt that other people are conspiring against you?*’										
4 Electrical influence	329 (48.0%)	238 (34.7%)	92 (13.4%)	26 (3.8%)	415 (60.6%)	198 (28.9%)	54 (7.9%)	18 (2.6%)	0.53	0.51
*‘Have you ever felt that electrical appliances, such as PCs, can affect your thoughts?*’										
5 Thought insertion	445 (65.0%)	189 (27.6%)	36 (5.3%)	15 (2.2%)	463 (67.6%)	145 (21.2%)	55 (8.0%)	22 (3.2%)	0.65	0.63
*‘Have you ever felt that the thoughts in your head are not your own?*'										
6 Thought broadcasting	431 (62.9%)	185 (27.0%)	53 (7.7%)	16 (2.3%)	485 (70.8%)	140 (20.4%)	45 (6.6%)	15 (2.2%)	0.65	0.63
*‘Have your thoughts sometimes been so vivid that you have been worried other people might hear them? Perceptual abnormalities’*										
7 External control	462 (67.4%)	175 (25.5%)	28 (4.1%)	20 (2.9%)	488 (71.2%)	118 (17.2%)	57 (8.3%)	22 (3.2%)	0.70	0.67
*‘Have you ever felt that there is another force outside of you who is in control of you?*’										
8 Auditory hallucinations	484 (70.7%)	170 (24.8%)	19 (2.8%)	12 (1.8%)	522 (76.2%)	114 (16.6%)	36 (5.3%)	13 (1.9%)	0.68	0.64
*‘Have you ever heard voices when you were completely alone (not radio or TV)?’*										
9 Visual hallucinations	574 (83.8%)	88 (12.8%)	15 (2.2%)	8 (1.2%)	582 (85.0%)	68 (9.9%)	28 (4.1%)	7 (1.0%)	0.62	0.58
*‘Have you ever seen objects, people, or animals that no one else can see?’*										

### 3.2 Sex Invariance of the CAPE‐9 Scale

We were able to show the invariance across sex at the configural, metric, and scalar levels (Table 3). No statistically significant difference was found between males and females in terms of CAPE‐9 scores (0.46 ± 0.41 vs. 0.48 ± 0.39, *t*(683) = −0.65, *p* = 0.516).

**TABLE 3:** Measurement invariance of the CAPE‐9 scale across sex and countries in the total sample.

Model	CFI	RMSEA	SRMR	Model Comparison	ΔCFI	ΔRMSEA	ΔSRMR
Model 1: Three‐factor model							
Configural	0.939	0.058	0.045				
Metric	0.939	0.055	0.050	Configural vs. metric	< 0.001	0.003	0.005
Scalar	0.928	0.055	0.051	Metric vs. scalar	0.011	< 0.001	0.001
Model 2: One‐factor model							
Configural	0.917	0.064	0.048				
Metric	0.914	0.061	0.056	Configural vs. metric	0.003	0.003	0.008
Scalar	0.903	0.061	0.057	Metric vs. scalar	0.011	< 0.001	0.001

Abbreviations: CFI, comparative fit index; RMSEA, Steiger‐Lind root mean square error of approximation; SRMR, standardised root mean square residual.

### 3.3 Convergent Validity of the CAPE‐9

Higher physical aggression (*r* = 0.24, *p* < 0.001), verbal aggression (*r* = 0.38, *p* < 0.001), anger (*r* = 0.33, *p* < 0.001), hostility (*r* = 0.38, *p* < 0.001), insomnia severity (*r* = 0.35, *p* < 0.001), anxiety (*r* = 0.43; *p* < 0.001), and depression (*r* = 0.47, *p* < 0.001) were significantly associated with higher CAPE‐9 scores.

## 4 Discussion

Despite its widely established psychometric qualities and well‐known clinical and research usefulness, the length of the CAPE‐P in its currently available Arabic version (20 items) may represent a potential limitation in data collection. Lengthy measures can affect data quality as they may negatively affect respondent engagement (Rammstedt and Beierlein 2014), whereas shortened versions may be more attractive to respond to whilst maintaining adequate psychometric qualities and the same level of information. This study proposes to validate a shorter, more practical, and user‐friendly 9‐item version of the CAPE‐P for use amongst the adult Arabic‐speaking population. Our results indicated acceptable fit values for both the single‐factor and three‐factor models of the Arabic CAPE‐9. In addition, the scale showed measurement invariance across sex, good internal consistency and concurrent validity, therefore suggesting its accuracy and suitability for use as a brief, time‐effective self‐report tool to capture PEs at the general population level.

Our results on the internal structure of the Arabic CAPE‐9 demonstrated that the overall model fit of the tridimensional factor structure (consisting of ‘Persecutory Ideation’, ‘Bizarre Experiences’, and ‘Perceptual abnormalities’ sub‐dimensions) was acceptable. In addition, a unidimensional model was tested and exhibited borderline fit indices, with all nine items loading onto one factor. These findings support previous work that suggested the use of a mean total score of the CAPE‐P, representing a single continuum of positive psychotic symptoms (Bukenaite et al. 2017; Sun et al. 2020). Although a considerable amount of previous evidence supported a three‐factor structure of the two short forms of the CAPE (Birkenæs et al. 2023; Capra et al. 2017; Núñez et al. 2015; Therman and Ziermans 2016), the adoption of the unidimensional model could be relevant for diagnostic purposes (Reininghaus et al. 2013). At the same time, adopting the tridimensional model could be potentially informative for discerning differential links between PEs and psychopathology, as it was, for example, shown that ideas of reference are more closely connected to the development of affective disorders and suspiciousness is more strongly linked to psychosis proneness (Unterrassner et al. 2017). Overall, and as currently recommended (Unterrassner 2018), we endorse the use of the three subtypes to estimate PEs levels in future clinical and research practises.

Internal consistency coefficients estimates were of 0.84 (alpha) and 0.84 (omega) for the total score, and ranged from 0.64 to 0.70 for both alpha and omega values for the three sub‐scores. This evidence showing good reliability of the Arabic CAPE‐9 further supports its suitability for capturing PEs in adults from the general population. These results mirror those of the initial psychometric validation of the CAPE‐9, which revealed a McDonald omega value of 0.74 for the CAPE‐9 total score in community male adults (Birkenæs et al. 2023). The results are also in line with other studies showing satisfactory reliability of the short 15‐item form of the CAPE‐P in various settings, including schools and universities (Capra et al. 2017; Núñez et al. 2015; Sun et al. 2020), primary care (Knight et al. 2020), and mental health services (Bukenaite et al. 2017). Furthermore, our current findings indicated that the structure of the Arabic CAPE‐9 had good model fit across the two sex groups and showed invariance between male and female adult participants. This further reinforces the good psychometric properties of the CAPE‐9, and implies that between‐sex comparisons can be performed meaningfully. Indeed, determining such a measurement equivalence allows inferring that differences found between males and females in CAPE‐9 scores should be considered as reflecting different degrees of PEs, and are not due to the scale measuring PEs differently depending on the respondent's sex. Finally, the pattern of correlations with external variables of the CAPE‐9 in its Arabic version build on and strengthen previous findings in the literature regarding the validity of the measure. Notably, and consistent with an extensive body of literature, significant positive associations were found between PEs and psychological distress (in the form of depression, anxiety and stress) (Fekih‐Romdhane, Jahrami, Away, et al. 2023; Kelleher, Keeley, et al. 2012; Sullivan et al. 2014), insomnia severity (Farah et al. 2023; Fekih‐Romdhane, Stambouli, et al. 2023; Fekih‐Romdhane, Hallit, et al. 2022; Fekih‐Romdhane, Lamloum, Loch, et al. 2023; Fekih‐Romdhane, Malaeb, Loch, et al. 2023), and the different types of aggression (physical, verbal, anger, hostility) (Fekih‐Romdhane, Hallit, et al. 2023; Fekih‐Romdhane, Malaeb, Loch, et al. 2023; Honings et al. 2016). Altogether, such findings and observations support and extend current knowledge on the robust psychometric properties and utility of the CAPE‐9 as an efficient measure of PEs. This further expands upon the previous evidence suggesting that PEs can be regarded as a risk marker for a broad array of behavioural problems and non‐psychotic psychopathology in young people from the general population (Fekih‐Romdhane 2024; Fekih‐Romdhane, Lamloum, Loch, et al. 2023; Fekih‐Romdhane, Malaeb, Loch, et al. 2023; Linscott and Van Os 2013; Rössler et al. 2007; Rubio et al. 2012).

### 4.1 Study Limitations

Before implications and conclusions can be drawn, it is important to acknowledge potential limitations of this study. The sample exclusively consisted of adults from the general population, which may limit the generalizability of findings. Therefore, the suitability of the measure for clinical samples and adolescents' populations still needs to be confirmed by further research. Moreover, the sample was predominantly composed of females and highly educated people, a characteristic frequently observed in online and convenience‐based mental health surveys. This may limit the extent to which the findings can be generalised to the broader adult population. Sex differences and education attainment may influence the perception, interpretation and reporting of PEs, particularly in self‐report measures, as higher mental health literacy and symptom awareness may lead to differential endorsement of items. Consequently, caution is warranted when extrapolating the results. Furthermore, whilst previous research has frequently supported a multidimensional structure of PEs, it is possible that sample characteristics contributed to the present findings. Prior findings suggest that ideas of reference are more closely associated with affective and internalising symptoms (Rodríguez‐Testal et al. 2019), which tend to be more prevalent amongst females (Keyes and Platt 2024). On the other hand, suspiciousness and paranoid ideation are more strongly linked to psychosis proneness (Wilcox et al. 2014), which is more commonly observed in males, particularly during adolescence and young adulthood (Zhu and Xue 2025). In this context, the relative underrepresentation of males may have influenced the variability of certain item domains, potentially favouring a unidimensional solution. In addition, only self‐report measures were adopted, and future studies using gold‐standard clinician‐rated interviews are required to examine the sensitivity and specificity of the CAPE‐9. Arabic‐speaking participants were gathered from one Arab country, and it is unknown whether our Arabic version of the CAPE‐9 is invariant and applicable across diverse Arab countries and cultural backgrounds. Future cross‐national studies are warranted to address this point. Test–retest reliability could not be tested and established in this study because of the cross‐sectional design.

### 4.2 Clinical and Research Implications

Our results add to previous evidence that the CAPE‐9 is valid, reliable, and suitable for use amongst community adults. Providing a brief, yet valid version of the CAPE in the Arab language will hopefully enable accurate, fast, and convenient measurement of PEs in Arab settings, which is essential for a timely and effective referral to specialised risk assessment, early management planning, and reduction of the incidence of psychotic and non‐psychotic outcomes in targeted communities. Indeed, as the CAPE‐9 is used as a first screening tool to detect young people at risk for psychotic disorders (Jaya et al. 2021), its implementation in routine clinical practise may improve recognition of a substantial proportion of undetected patients with psychotic disorders (Boonstra et al. 2009) and contribute to reducing the very long duration of untreated psychosis still observed in Arab countries. It is important to highlight that the CAPE‐9 is not a diagnostic tool, but can only serve as an initial screening measure to be followed–when warranted– by structured diagnostic interviews. The use of the three‐factor model is recommended in future clinical and research applications of the scale, as it may be highly informative for various assessment objectives.

## 5 Conclusions

Due to scanty research on psychosis detection and prevention in the Arab world, and recognising the need for a briefer version of the CAPE to increase its applicability in clinical and research contexts, our research aimed at validating the CAPE‐9 into the Arabic language. The CAPE‐9 showed appropriate psychometric properties in terms of validity and reliability. It also revealed invariance across sex, which enables meaningful comparisons between males and females. Overall, findings suggest that the Arabic CAPE‐9 is a useful tool for screening PEs in general population individuals. As a short, simple, economic, and convenient‐to‐administer measure of PEs, the CAPE‐9 is amenable to widespread use. It has, therefore, the potential to foster research and clinical practise by easing data collection, lessening burden and enhancing engagement of respondents.

## Funding

The authors have nothing to report.

## Ethics Statement

Ethics approval for this study was obtained from the ethics committee of the School of Pharmacy at the Lebanese International University.

## Consent

Written informed consent was obtained from all subjects; the online submission of the soft copy was considered equivalent to receiving written informed consent. All methods were performed in accordance with the relevant guidelines and regulations.

## Conflicts of Interest

The authors declare no conflicts of interest.

## Data Availability

The data that support the findings of this study are available on request from the corresponding author. The data are not publicly available due to privacy or ethical restrictions.
